# Effects of survey administration mode on response profiles are predictable, and robust across countries: Evidence from 29 countries using machine-learning models

**DOI:** 10.1371/journal.pone.0330182

**Published:** 2025-09-12

**Authors:** Allon Vishkin, Eddie Bkheet

**Affiliations:** Faculty of Data and Decision Sciences, Technion—Israel Institute of Technology, Haifa, Israel; University of Naples Federico II: Universita degli Studi di Napoli Federico II, ITALY

## Abstract

Survey data collection can be administered in different modes, including face-to-face interviews and self-completion modes such as paper-and-pencil or web-based surveys. Do data collected in these different modes reliably differ across countries? We address this question using responses from 145,361 respondents in 29 countries to 46 questions in Rounds 8–10 of the European Social Survey (ESS), a large-scale social research project that conducts cross-national surveys in Europe and whose data has been used in thousands of publications. The ESS is typically administered in face-to-face interviews, but due to the COVID-19 pandemic data from nine countries in the tenth round were collected using self-completion methods. In line with previous findings demonstrating differences between administration modes, we show that machine-learning models can predict how surveys were administered, suggesting that data collected in the different modes are not comparable. More critically, we show that even when these models are trained on data from a set of countries, they can predict how surveys were administered in a completely novel country, which indicates that responses in different administration modes reliably differ across countries. Finally, we investigate extreme response styles as one difference in the response profiles of the two different modes. In addition to addressing concerns of data comparability in the ESS, these findings reveal that administration modes of surveys lead to reliable cross-national differences in response profiles.

## Introduction

Surveys can be administered in various modes, including in interviews (whether in-person or telephone), or by self-completion (whether online or paper-and-pencil). Numerous methodological studies have demonstrated that response profiles across these various modes are not necessarily equivalent [1–10]. By response profiles, we mean the overall pattern or combination of responses that individuals provide across a set of survey items. The current investigation leverages a recent change in the administration mode of the European Social Survey as a natural experiment to examine differences in response profiles using machine learning models. Specifically, the current investigation examines whether response profiles in different administration modes are or are not reliably different, whether these differences in response profiles generalize across countries, and to what extent these differences may be attributable to more extreme response styles.

### Response profile differences between administration modes

Several methodological studies have examined whether and how response profiles differ between administration modes. One line of work has examined how administration modes affect socially desirable responding [2,4,11,12], which refers to the tendency to provide responses that survey respondents deem will be viewed favorably by others. A finding from this literature is that social desirability effects are stronger in interviewer administration modes than in self-administration modes, likely due to a greater need to be viewed favorably in the eyes of the interviewer. This leads people in an interview administration mode to report lower rates of behaviors perceived as inappropriate, as well as higher rates of behaviors perceived as appropriate. For instance, in interview administration modes, people report lower illicit drug use, but report higher religious service attendance [13]. Another line of work has examined whether primacy or recency effects are more common in certain administration modes [11,14–16]. A conclusion from this line of work is that when questions are presented orally in interview modes, the limited ability to process early response options leads to a recency effect [3]. Meanwhile, when questions are presented visually in self-completion modes, early responses undergo greater cognitive processing and therefore lead to primacy effects. Relatedly, a line of work has examined whether response profiles in interviews, versus self-completion, are more differentiated [1] or extreme [3]. One finding is that responses are more extreme in interview modes, although this appears to depend on the valence of the items [3].

The implications of how administration modes affect response profiles are particularly relevant to large and highly cited cross-national surveys, such as the World Values Survey and the European Social Survey (ESS). Data from each of these surveys have been used in thousands of publications – for instance, a search in google scholar for “data from the European Social Survey” generated 5,660 results in February 2025 – with data frequently aggregated across rounds of administration given their ex-ante harmonization [17] across locations and rounds. In the 10^th^ round of the ESS, administered during the COVID-19 pandemic in which in-person interviews were not feasible in many locations, self-completion administration was used in some countries in place of the traditional interview administration. This change in mode presents a unique opportunity to test whether response profiles are affected by administration modes, and, if they are affected, whether these effects are consistent or variable across countries.

Previous studies examining the effect of administration modes have been conducted by The Core Scientific Team of the ESS, but these have been smaller in scope. These studies have been typically implemented as supplements to the regular administration of the ESS in up to four countries [5]. Three studies conducted between 2003–2011 investigated the effect of administration mode on measurement, but these were never conducted in more than two countries. Three additional studies conducted between 2006–2012 investigated the practical challenges of conducting the ESS in more than one administration mode, such as differing response rates as a function of administration mode and survey length. None of these studies included the administration of a full-length survey of the ESS on full samples across several countries. For instance, one study included the administration of the ESS via the standard mode as well as via telephone in four countries. Telephone samples in the four countries ranged from 83-369 respondents – significantly smaller than by-country samples sizes in the ESS. Additional studies were conducted under the ESS in response to the COVID-19 pandemic, which limited the ability to conduct face-to-face interviews in some countries due to social distancing restrictions. One study conducted in 2020 examined web- and paper-based self-completion modes in three countries on a subset of the ESS [18]. Several additional studies were conducted in one or two countries, including a study conducted in 2021 examining response rates to self-completion modes as a function of survey length [19] and a study conducted in 2021–2022 on different incentive schemes for self-completion modes [20]. Thus, while previous studies have examined the effect of administration modes, they have been limited in scope by including a limited set of countries, a limited set of participants, or a limited set of questions.

The literature demonstrating that administration modes affect response profiles may suggest that the common practice of combining data across rounds of the ESS might not be recommended with self-completion data in Round 10. However, much of the previous work on administration modes is not directly relevant to the administration of the ESS. The methodology of the interview mode of the ESS requires visually presenting to respondents the responses using showcards for most questions. Thus, the suggestion that audio presentation in interview mode increases recency effects [3] is not directly relevant to the administration mode change in the ESS. Furthermore, while some of the questions in the ESS may elicit some social desirability concerns, such as questions on political attitudes, many or most of the questions in the ESS do not address questions which may elicit strong social desirability concerns, such as use of illicit drugs. Finally, and most critically, the vast majority of the experimental research on the effects of administration modes has been conducted in 4–5 countries at the most [5,7]. Thus, there is little research examining whether different administration modes lead to broader differences in response profiles. For instance, while response styles may be more extreme in interview modes [3], it is unclear whether this appears universally across countries.

## The present investigation

This investigation uses machine learning models, whose use and application for understanding survey data has grown in recent years [21]. The purpose of this investigation is three-fold. First, this investigation tests whether, and to what extent, response profiles vary by administration modes. The first research question is the following:

RQ1: Do respondents’ response profiles differ based on the survey’s administration mode?

As summarized above, much research has established that response profiles do vary by administration mode, whether due to desirability effects, primacy or recency effects, or extreme response styles. However, previous data was not on the same scale as the data in the current investigation. Based on the previous literature, we predicted that machine learning models will be able to accurately classify respondents’ administration mode based on their response profile. We had no expectations regarding the magnitude of this effect, such as whether accuracy will be slightly above chance or whether it will be above 90%. To address this research question, we test whether a machine learning model can predict whether responses came from a group of respondents who completed the survey face-to-face, or from a group of respondents who completed the survey using self-completion methods (paper-and-pencil or web-based).

Assuming the predicted findings for RQ1 are obtained, the second purpose of this investigation is to establish whether the administration-dependent response profiles generalize across countries. The second research question is the following:

RQ2: Are the differences in response profiles which are due to administration mode equivalent across countries?

As summarized previously, most studies on the effect of administration mode on response profiles have been conducted in a single country. Some previous findings may be country-dependent. For instance, the effects of administration on socially desirable responding may be more common in more collectivist countries, whether the social judgment of others may carry more weight [22,23]. Thus, the manner in which administration modes influence response profiles may vary between countries. Given the lack of prior work on how administration mode affects response profiles across countries, we had no a priori predictions regarding this research question. We tested this research question by training a machine learning model to classify responses based on their administration mode using data from a given set of countries, and then tested whether this model can predict the administration mode of respondents in a completely novel country.

Assuming the predicted findings for RQ1 are obtained, the third purpose of this investigation is to examine extreme response styles as one possible difference in the response profiles of the two different modes. The third research question is the following:

RQ3: Are the differences in response profiles which are due to administration mode attributable to differences in extreme response styles?

We focused on the effect of administration mode on extreme response styles because it is easy to define and has been investigated previously, with mixed findings [3,5]. Other types of response styles, such as primacy or recency, are less relevant in the present comparison, given that response options for many questions in the interview format were presented on showcards. Meanwhile, socially desirable responding is more difficult to operationalize given that what is socially desirable varies from one society to another. The machine learning models enabled us to test the contribution of extreme response styles with relative ease. In particular, we tested how the accuracy of the models is affected when the values of all variables were recoded as the absolute distance from the mid-point of the scale, as well as when adding these recoded values as additional features to the model.

A concern common to the evaluation of all the research questions is that the administration mode in Round 10 is fully confounded with respondents’ country of origin. In other words, the administration mode in which respondents completed Round 10 was fully determined by their country, with nine countries adopting a self-completion mode and all other countries maintaining an interview mode. Thus, in no country was there more than one type of administration mode in Round 10. Consequently, any machine learning analysis between different administration modes within Round 10 might be learning to classify respondents by country of origin rather than by administration mode. To address this confound, the main analysis was between the 9 countries with a self-completion mode in Round 10, versus those same countries in previous rounds. However, the samples with self-completion modes in Round 10 had notably larger samples than samples with interview modes. Consequently, we compared data in Round 10 to data from two of the previous rounds: Round 8 (administered in 2018) and Round 9 (administered in 2016). However, this introduces a confound of time: differences between the two administration modes may not be driven by the administration mode, but by the later time of Round 10 (compared to Rounds 8 and 9). To address this, we captured the contribution of time to the predictive power of the model by running a separate model comparing the 20 countries which had an interview administration mode in Round 10, versus those same 20 countries in Rounds 8 and 9.

## Method

### Sample

Data were analyzed from the ESS, Rounds 8–10. Round 10 of the ESS contains samples from 31 countries. Of these, two had no data from Rounds 8 or 9 (Greece and North Macedonia) and therefore were not included in the analyses. Of the remaining countries, 9 used a novel self-completion mode (total respondent *N* = 22,074) and 20 used the standard interview mode (total respondent *N* = 33,383). We also selected respondents from these 29 countries who participated in Rounds 8 or 9 (*N* = 25,928 in the 9 countries with a self-completion mode in Round 10; *N* = 63,976 in the 20 countries with an interview mode in Round 10). Henceforth, we refer to these four classes as follows:

*Class SC-10*: Respondents who completed Round 10 in a self-completion mode (9 countries).
*Class* SC-89: Respondents in Rounds 8-9 originating from the nine countries for which a self-completion mode was used in Round 10 (but who themselves responded in an interview mode).
*Class IV-10*: Respondents who completed Round 10 in an interview mode (20 countries).
*Class IV-89*: Respondents in Rounds 8-9 originating from the 20 countries for which an interview mode was used in Round 10 (and who themselves responded in an interview mode).

See Table 1 for a breakdown of countries and samples in each mode.

**Table 1:** Respondents in each mode and each Round by country.

Mode	Country	Abbreviations	*N* Round 10	*N* Round 8 + 9
			*Class SC-10*	*Class SC-89*
*Self-completion mode in Round 10*	Austria	AT	2003	4509
	Cyprus	CY	875	781
	Germany	DE	8725	5210
	Israel	IL	1308	2557
	Spain	ES	2283	3626
	Latvia	LV	1023	918
	Poland	PL	2065	3194
	Serbia	RS	1505	2043
	Sweden	SE	2287	3090
	*Total*		22,074	25,928
			*Class IV-10*	*Class IV-89*
*Interview mode in all rounds*	Belgium	BE	1341	3533
	Bulgaria	BG	2718	2198
	Croatia	HR	1592	1810
	Czechia	CZ	2476	4667
	Estonia	EE	1542	3923
	Finland	Fi	1577	3680
	France	FR	1977	4080
	Hungary	HU	1849	3275
	Iceland	IS	903	1741
	Ireland	IE	1770	4973
	Italy	IT	2640	5371
	Lithuania	LT	1659	3957
	Montenegro	ME	1278	1200
	Netherlands	NL	1470	3354
	Norway	NO	1411	2951
	Portugal	PT	1838	2325
	Slovakia	SK	1418	1083
	Slovenia	SI	1252	2625
	Switzerland	CH	1523	3067
	United Kingdom	UK	1149	4163
	*Total*		33,383	63,976

### Variables

Each round of the ESS contains variables common to all the rounds, as well as variables unique to a particular round or subset of rounds. We identified relevant variables based on several criteria. First, we excluded variables that did not appear in all three rounds. Second, since we sought to run the analyses above and beyond particular countries, we excluded variables that were country-specific (e.g., variable *prtvtcat*, administered only in Austria and assessing which party the participant last voted for). This left in variables typically considered as part of the ESS core questionnaire. Third, since we expected that the administration mode would influence the profile of responses on continuous or ordinal scales, we excluded categorical variables or variables with binary responses. This left in 50 variables. Of these variables, two variables (*imsmetn* and *imdfetn*) were omitted in one country in Round 9, leading to a potential confound between missing data and administration mode. For two further variables (*wkdcorga* and *iorgact*), respondents answered them only if a response to a previous question indicated these questions were relevant to them. In such a case, it would make little sense to imputing values for missing responses. This left 46 variables (as well as country and ESS round) on which the analyses were conducted.

### Imputing missing data

Overall, 3.40% of the values were missing in our dataset spread across 67.17% of the respondents (class SC-10: 2.78% values across 59.84% of respondents; class SC-89: 3.68% values across 67.70% of respondents; class IV-10: 3.40% values across 70.50% of respondents; class IV89: 3.49% values across 67.75% of respondents). We note that these rates of missing data are significantly lower than in previous published studies in psychological studies, where they can be as high as 75% (Savani et al., 2020).

We considered three methods for dealing with missing data: mean value imputation, KNN imputation, and the built-in method of our chosen machine-learning algorithm [24]. The performance of each method was evaluated by training models using those imputation strategies and comparing their performance on a validation dataset. We found that the built-in method of the machine-learning algorithm produces the best results on the validation set and therefore used this method. This method treats missing values as a separate branch in decision trees, which avoids biases from ad-hoc imputation [25].

### Splitting the data

We split the data into two parts. For the analyses across countries, we selected 80% of observations in each country (the seen data) for the model-building and training phase such that the proportion of each of the two target classes is approximately the same as in the complete set. The seen data was further split into 80% for training and 20% for validation (64% and 16% of the entire dataset, respectively). The remaining 20% of the data (the unseen data) were used to test the predictions of the model. For the analyses within countries, separate models were run for each country, with 80% of observations in each country comprising the seen data and 20% comprising the unseen data. For the analyses testing generalizability to a novel country, all countries but one comprised the seen data, and the remaining country comprised the unseen data.

### Machine learning model and analyses

**Fig 1:**
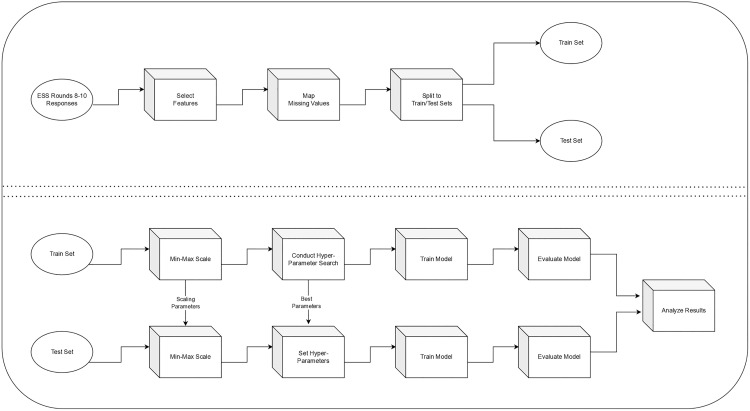
Depiction of the machine learning procedure. The workflow illustrates the sequential steps used in the analysis, including data preprocessing, feature extraction, model training, validation, and evaluation. Arrows indicate the steps of the procedure and the iterative process of model refinement.

The procedure we used for the machine learning analysis is illustrated in Fig 1. We used a desktop computer running Linux with a 48 core CPU, 995 GB RAM, and 2 Nvidia RTX A6000 48GB graphics cards. Analyses were conducted in the python 3.8 environment. The script and final model are available in the OSF data repository for this project: https://osf.io/t73kv/?view_only=a8be0616d0bc42c982dfd281a98308f0.

We used as our machine learning algorithm LightGBM, an efficient gradient boosting decision tree algorithm [24]. To improve its efficiency, it uses Gradient-based One-Side Sampling (GOSS) and Exclusive Feature Bundling (EFB). GOSS excludes a substantial portion of data instances characterized by low gradients, and uses the remaining data for estimating information gain. EFB bundles mutually exclusive features to reduce the number of features in the model. It has been demonstrated that LightGBM speeds up the training process of conventional gradient-based decision tree algorithms while achieving nearly the same accuracy [24]. LightGBM performed binary classification on the task of predicting the administration mode of survey responses. Specifically, we trained a LightGBM model on a dataset of survey responses, where each response was labeled as either from Round 10 or from Rounds 8–9. We used the responses to the 46 survey questions as features to train the model.

In a categorization task with imbalanced sample sizes between two classes (i.e., chance level of correct categorization is not equal to 50%), the categorization accuracy can be a misleading representation of a model’s performance. A more accurate representation of the model performance is often assessed using a statistical measure called the F1 score. An F1 score combines both the precision of a model (the number of true positive [TP] results divided by the number of true positive results and false positive [FP] results) and the recall of a model (the number of true positive results divided by the number of all instances that should have been identified as positive – true positive results and false negative [FN] results). The equation for an F1 score is as follows:


$$F1=\frac{2\times TP}{2\times TP+FN+FP}$$

Since all our models have imbalanced sample sizes, we report both the categorization accuracy and F1 score of all models.

## Results

### Predictability of administration mode across all countries

First, we evaluated RQ1 regarding whether respondents’ response profiles differ based on the surveys’ administration mode. In particular, we tested whether our machine learning model can categorize whether respondents in the unseen data belonged to classes *SC-10* or *SC-89* above random accuracy. Given the imbalanced sample sizes of the two classes, the chance level of selecting the correct administration mode is 54.5%. Meanwhile, our model had an accuracy of 77.6% in categorizing respondents in the unseen data, substantially higher than the chance level. The F1 score, which accounts for imbalanced sample sizes as we described above, was 75.0%, which indicates a good balance between precision and recall. Feature importance for each of the 46 variables based on gain (the contribution of a feature to the model’s prediction) appear in S1 Fig of the Supplementary Materials.

These metrics indicate that the response profiles in the different administration modes of classes *SC-10* and *SC-89* are sufficiently distinct to be detected by a machine-learning model. However, the two classes differ not only in their administration mode, but also by their rounds. To examine whether the model’s success in categorization reflect differences in rounds rather than their administration, we ran a new model on the classes *IV-10* and *IV-89*, which have identical administration modes but differ in their rounds. The model was trained to classify if the response came from round 10 or not. Given the imbalanced sample sizes, the random accuracy of selecting the appropriate categorization was 65.6%. Meanwhile, the model had an accuracy of 69.4% in categorizing respondents in the unseen data, which is not substantially higher than the random accuracy, and lower than in the categorization task of the previous model. Furthermore, the F1 score was very low at 34.5%. An examination of the results revealed that this is driven by a high rate of false negatives. Thus, the previous model’s ability to categorize respondents between classes *SC-10* and *SC-89* appears to be based on their differences in administration modes and not their differences in rounds.

### Predictability of administration mode per country

**Fig 2:**
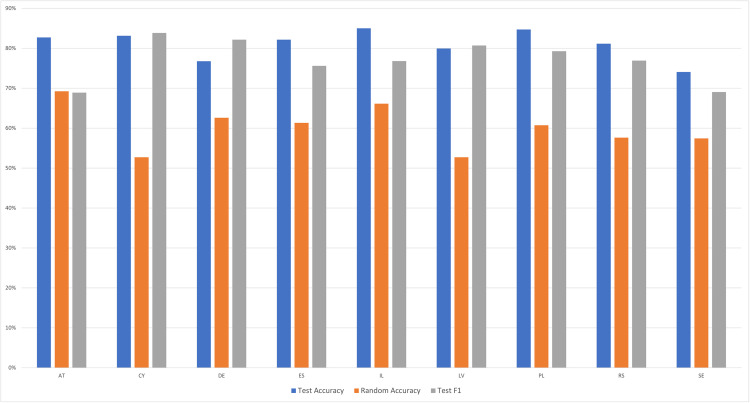
Metrics for per-country models among the countries with different administration modes between Rounds 10 and Rounds 8-9. The figure presents model performance across countries that experienced a change in survey administration mode between rounds, using metrics of test accuracy and F1-score relative to random accuracy. See Table 1 for a key to country abbreviations.

These findings are in line with our prediction that a machine learning model will be able to accurately classify the administration mode of respondents based on their response profiles. However, the preceding models ignored the nesting of respondents in particular countries. Specifically, sample sizes were not equivalent across countries and Rounds, and differed by up to an order of magnitude, which might have introduced a confound into the previous results. For instance, the largest sample of country-per-round was Germany in Round 10 (see Table 1). Germany, in Round 10, used the novel self-completion mode, and so the model may be particularly good at categorizing respondents in the self-completion mode vs. interview mode due mostly to the large sample of German respondents. To address any concerns about whether and how country differences in sample sizes may have biased results, we ran new models with each country on its own, for each of the nine countries with respondents in the *SC-10* and *SC-89* classes. Across the nine countries, the average random accuracy was 60.1%. Meanwhile, the average model accuracy was 81.1%, and the average F1 score was 77.0%. Results in Fig 2 illustrate that in every country with a different administration mode, a model was successful in its categorization task above and beyond chance level.

**Fig 3:**
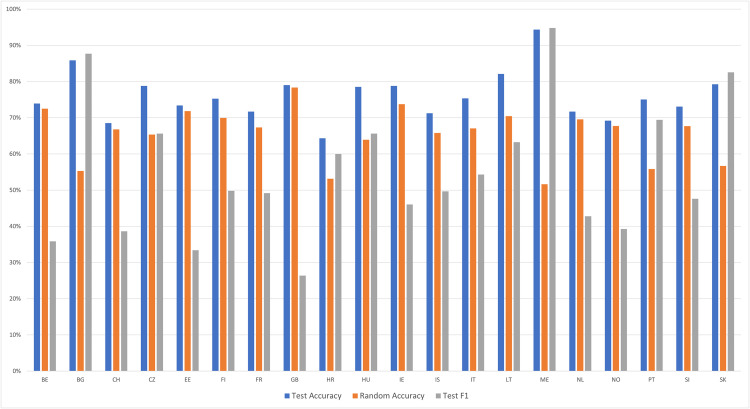
Metrics for per-country models among the countries with identical administration modes between Rounds 10 and Rounds 8-9. The figure presents model performance across countries that experienced no change in survey administration mode between rounds, using metrics of test accuracy and F1-score relative to random accuracy. See Table 1 for a key to country abbreviations.

Next, we evaluated whether the same pattern of findings emerges when categorizing respondents from the 20 countries in classes *IV-10* and *IV-89*, which differ by rounds but have identical administration modes. If results do generalize, that would suggest that the per-country analyses capture differences in rounds rather than differences in administration modes. Across the 20 countries, the random accuracy was 65.5%. The average model accuracy was 76.0%, and the average F1 score was 55.1%. Results in Fig 3 reveal that the model accuracy was greater than the random accuracy by less than 6 percentage points in 11 of 20 countries, and F1 scores were poor in a majority of countries. Thus, model performance for per-country categorization between classes *IV-10* and *IV-89* was poorer than for the categorization between classes *SC-10* and *SC-89.* Overall, these findings reveal that even when running per-country models, respondents are correctly categorized by their administration mode above chance, and this is only partially attributable to their different rounds.

### Generalizing to a novel country

**Fig 4:**
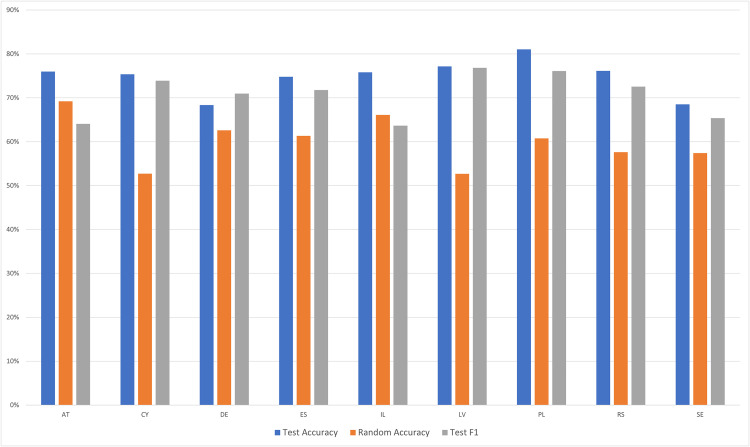
Metrics for models with all data from a given country in the unseen data, for countries with differing administration modes. The figure presents model performance across countries that experienced a change in survey administration mode between rounds, using metrics of test accuracy and F1-score relative to random accuracy, when all of a given country’s data was in the unseen data and the model was trained on the remaining countries. See Table 1 for a key to country abbreviations.

Next, we evaluated RQ2 regarding whether differences in response profiles by administration mode are equivalent across nations. In all preceding analyses, data from a given country always appeared both in the seen data and in the unseen data. If there are reliable differences in the responses to different administration modes, a model should be able to categorize respondents based on their administration mode even when they originate from a novel country. To test this question, we trained a model on 8 of the 9 countries with different administration modes (classes *SC-10* and *SC-89)*, and tasked it with categorizing respondents from a ninth country. This was repeated with a new model for each of the 9 countries. The average random accuracy was 60.1%. Meanwhile, the average model accuracy was 74.8%, and the average F1 score was 70.6%. Results in Fig 4 illustrate high model accuracy and a comparable F1 score in 7 of the 9 countries.

**Fig 5:**
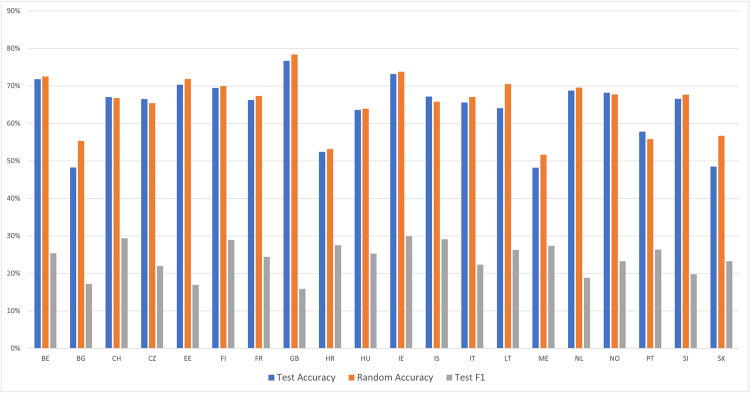
Metrics for models with all data from a given country in the unseen data, for countries with identical administration modes. The figure presents model performance across countries that experienced no change in survey administration mode between rounds, using metrics of test accuracy and F1-score relative to random accuracy, when all of a given country’s data was in the unseen data and the model was trained on the remaining countries. See Table 1 for a key to country abbreviations.

Next, we examined whether the ability to generalize to a novel country also occurs among countries with the same administration mode. In particular, among the 20 countries with the same administration mode in Rounds 8–9 and 10 (classes *IV-10* and *IV-89*), we taught the model to classify respondents from 19 countries whether they completed the survey in Rounds 8–9 or in Round 10. Then, we tested the accuracy of the model in classifying respondents to Rounds 8–9 or Round 10 in the remaining country. We repeated this procedure for each country. The random accuracy was 65.5%. Meanwhile, the average model accuracy was slightly lower at 64.0%, and the average F1 score was 24.0%. Results in Fig 5 illustrate that model accuracy was typically no greater than random accuracy, and F1 scores were consistently poor.

Overall, these findings show that respondents are categorized based on their administration mode above chance even in novel countries, and this is not attributable to differences in the rounds of the administration modes. This suggests that the effect of different administration modes on respondents’ response profiles is consistent across respondents from different countries. As a further test of this, we compared feature importance, or the contribution of a feature to the model’s prediction, in the main model vs. the country-specific models. Specifically, we compared feature importance in the main model tasked with classifying respondents based on the different administration modes (for classes *SC-10* vs. *SC-89*; see S1 Fig in the Supplemental Materials), with the feature importance in the nine models conducted on each country on its own for the same classes (as presented in Fig 2; for model-specific feature importance see S2–S10 Figs). Spearman’s rank-order correlation revealed a high degree of consistency between the rank order of features in the main model and the rank order of features in the country-specific models (Austria: *ρ* = .54, *p* < 0.001; Cyprus: *ρ* = .43, *p* = 0.004; Germany: *ρ* = .65, *p* < 0.001; Spain: *ρ* = .60, *p* < 0.001; Israel: *ρ* = .48, *p* < 0.001; Latvia: *ρ* = .74, *p* < 0.001; Poland: *ρ* = .61, *p* < 0.001; Serbia: *ρ* = .65, *p* < 0.001; Sweden: *ρ* = .68, *p* < 0.001), providing another indication that administration mode effects on response profiles have a high degree of consistency across countries.

### Extreme values by administration mode

Finally, we evaluated RQ3 regarding the possibility that the differing response profiles by administration mode are due to differences in extreme response styles. The preceding findings demonstrated across several different analyses that the response profiles of respondents to different administration modes of the same survey questions differ reliably enough to be detected by machine learning models. However, like many machine learning models, its decision-making processes are opaque. It is thus unclear what elements of mode-dependent response profiles drive the models’ ability to categorize responses. One possibility discussed earlier is that answers might be more extreme in one administration mode than in another administration mode. As an initial test of this possibility, we summed the number of times each respondent used the most extreme answer choice [26]. Extreme responses were more common in the self-completion mode in Round 10 (class *SC-10*; *M* = 10.53; *SD* = 6.46), relative to both their comparison group in Rounds 8–9 (class *SC-89*; *M* = 9.99; *SD* = 5.73) and the interview mode in Round 10 (class *IV-10*; *M* = 9.84; *SD* = 5.70). The effects are small, but may reflect a larger difference in how the entire scale ranges are used. To test whether this difference in scale use might have been used by the model to categorize responses based on administration modes, we replaced each value in the dataset with its distance from the middle value of the question. For example, if a question has a range of answers between 0 and 10, and a respondent’s answer was 3, it was replaced with 2 according to the following equation:


$$|\frac{\left(Min+Max\right)}{2}-Val|=|\frac{\left(0+10\right)}{2}-3|=2$$

In other words, these values reflect the absolute distance from the middle of the scale. The only exception was the variable *nwspol* because its values weren’t bounded. We used the mean instead of $\frac{\left(Min+Max\right)}{2}$. We used these recoded variables to run the same conceptual model as the first model in the analysis, with the task of categorizing responses from classes SC-10 and SC-89 across all countries.

As with the first model run in the analysis, random accuracy was 54.5%. Meanwhile, the test accuracy was 73.0% and the F1 score was 69.7% (see S11 Fig for feature importance). Both were slightly lower than the metric of the first model in the analysis where a classification task was conducted on class *SC-10* vs. *SC-89* (77.6% and 75.0%, respectively). However, we note the tremendous loss of data specificity when coding the absolute distance from the mean, since scales are collapsed in two – and despite this loss of data specificity, model accuracy remained comparable to that of the original model. Consequently, we interpret these results as a strong indication that extreme response styles likely play an important role in the successful categorization of responses as self-completion or interview in our models. As a further test of this, we ran a new model with two sets of features: the original variables, and their recorded absolute distances from the mean. Model accuracy was 77.1% and F1 score was 74.5% – very near the metrics of the first model of the analysis. Apparently, the recoded variables lose their predictive power when included with the raw variables, despite having high predictive power on their own. We interpret this result as indicating that much of the successful categorization of responses between the classes *SC-10* and *SC-89* might have relied on the more extreme response styles of the former. In support of this interpretation, an analysis of feature importance revealed that the order of feature importance remains relatively consistent in the model with the original scales (S1 Fig in the Supplemental Materials), versus when scales have been recomputed to reflect absolute distances (S11 Fig in the Supplemental Materials), *ρ* = .69, *p* < 0.001.

## General discussion

The present investigation leveraged an unplanned change to the administration of a large-scale cross-national survey due to the COVID-19 pandemic to test whether and how response profiles vary by administration mode. First, across countries, a machine learning model was successful in classifying respondents’ survey administration mode based on their responses to 46 questions. The accuracy of this model was not perfect but nevertheless substantial, with a chance level of 54.5% and accuracy of 77.6% - nearly halfway to complete accuracy. An additional model demonstrated that this is only partially attributable to differences in the rounds of the administration modes. Furthermore, additional models per country revealed that this was not attributable to the different by-country sample sizes. Thus, findings supported the prediction for RQ1 that respondents’ response profiles differ based on a survey’s administration mode.

Next, we tested whether these differences generalize across countries. We found that machine learning models that were trained on a set of countries to classify respondents’ administration mode were successful in classifying respondents’ administration mode in a novel country, based on both level of accuracy (in all 9 countries) and F1 scores (in 7 of 9 countries). Additional models demonstrated that this is only partially attributable to differences in the rounds of the administration modes. Furthermore, the rank order of feature importance was highly correlated across the different per-country models. These findings addressed RQ2 by demonstrating that the effect of administration modes on response profiles are, at least to a certain extent, equivalent and universal within the set of countries tested.

Finally, we tested whether the administration mode may have affected response profiles by influencing extreme response styles. First, we reduced the precision of the data used to train the models by recomputing values to reflect the absolute distance from the middle of the scale. Despite this loss of data, reduction in the accuracy of the model was minimal, suggesting that absolute distance from the middle of the scale is sufficient for it to classify the administration mode of respondents. Next, when we combined the original features and absolute-distance features, the accuracy of the model was not substantially greater, indicating that the information on the absolute distance from the mean is sufficient for the classification task, and adds no new information. Furthermore, the rank order of feature importance was highly correlated between the main model and the model with features coded as absolute distance. These findings addressed RQ3 by indicating that the differences in response profiles which are due to administration mode are almost entirely attributable to differences in extreme response styles.

### Limitations and future directions

The present investigation leveraged an unplanned change to the ESS to test response profile differences based on administration mode. Since no countries used more than one administration mode in a given round, a challenge we faced was demonstrating that the models were classifying respondents based on their administration modes and not the different rounds. We addressed this by demonstrating that in data from countries that differed only in their rounds, and not in their administration mode, model accuracy was substantially lower. Nevertheless, countries in which face-to-face interviews were not possible to conduct during the COVID-19 pandemic – and therefore had to opt for a self-completion method – may have experienced the pandemic more severely. Thus, differences between rounds cannot be fully ruled out as an alternative explanation to our findings. Furthermore, there are likely sample composition effects between the two modes, reflecting whether participants agree to complete the survey and thus affecting the composition of respondents in each mode [27]. Future work can investigate these questions using a random allocation to administration mode. One relevant data source is a within-country experiment in conducted under the ESS in two countries (Finland and Great Britain) where respondents were randomly assigned to one of two modes [28]. In addition, Round 12 of the ESS will include a random allocation of respondents between the two modes within countries [29], allowing for a more direct comparison.

Several studies have demonstrated that respondents from different cultural backgrounds vary in their response styles. For instance, extreme response styles have been found to vary across countries [30–32], as have acquiescent response styles and socially desirable responding [23]. The present investigation found that the effect of administration mode on response profiles is consistent across countries, even as it identified more or less extreme response styles as an outcome of the two different administration modes. These two sets of findings are not necessarily inconsistent: respondents from different countries may vary in the frequency with which they provide extreme responses, but may be similarly influenced by the extent to which different administration modes affect extreme responses. Even so, the samples in the present investigation originated from a single geographic region, whereas cultural differences in response styles have been demonstrated between more geographically distant regions (e.g., the Japan and Taiwan vs. Canada and the United States; [31]). Future work from more diverse regions is needed to establish whether the effects of administration modes on response profiles are consistent across countries.

While the present investigation focused on the comparison between interview vs. self-completion modes, further comparisons can be run to compare paper-and-pencil vs. web-based formats. In particular, the nine countries with a self-completion mode in round 10 had both formats available. Such a comparison would need to account for other concerns not present in the current investigation, such as self-selection into a particular format. In addition, the sample sizes within countries differ greatly between the two formats, sometimes at a ratio of more than 4:1. Nevertheless, such an investigation could be informative regarding whether or not there are substantive differences between the two different self-completion formats.

## Conclusion

The present investigation reveals that survey response profiles vary by administration mode, a difference that persists across multiple countries, indicating a robust and universal pattern. Machine learning models accurately classified respondents’ administration modes based on their response profiles, suggesting that data collected via different methods are not comparable. These differences, attributable in part to variations in extreme response styles, highlight the importance of considering administration mode in survey design and analysis to ensure data reliability and validity. Future research should further explore these findings’ implications for cross-national survey methodologies, such as by using randomized administration modes to isolate the effects of the mode from other confounding factors, as is planned for the upcoming ESS Round 12 [29].

## Supporting information

S1 Table Variables used in study. (DOCX)

S1 Fig Feature importance based on gain, for the classification task of classes SC-10 vs. SC-89. (DOCX)

S2 Fig Feature importance based on gain, for the classification task of classes SC-10 vs. SC-89, within Austria. (DOCX)

S3 Fig Feature importance based on gain, for the classification task of classes SC-10 vs. SC-89, within Cyprus. (DOCX)

S4 Fig Feature importance based on gain, for the classification task of classes SC-10 vs. SC-89, within Germany. (DOCX)

S5 Fig Feature Importance based on gain, for the classification task of classes SC-10 vs. SC-89, within Spain. (DOCX)

S6 Fig Feature importance based on gain, for the classification task of classes SC-10 vs. SC-89, within Israel. (DOCX)

S7 Fig Feature importance based on gain, for the classification task of classes SC-10 vs. SC-89, within Latvia. (DOCX)

S8 Fig Feature importance based on gain, for the classification task of classes SC-10 vs. SC-89, within Poland. (DOCX)

S9 Fig Feature importance based on gain, for the classification task of classes SC-10 vs. SC-89, within Serbia. (DOCX)

S10 Fig Feature importance based on gain, for the classification task of classes SC-10 vs. SC-89, within Sweden. (DOCX)

S11 Fig Feature importance based on gain, for the classification task of classes SC-10 vs. SC-89 after conversion of variables to absolute distance scores. (DOCX)

## References

[1] Fricker S, Galesic M, Tourangeau R, Yan T. An experimental comparison of web and telephone surveys. Public Opin Q. 2005;69: 370–92.

[2] Kreuter F, Presser S, Tourangeau R. Social Desirability Bias in CATI, IVR, and Web Surveys: The Effects of Mode and Question Sensitivity. Public Opin Q. 2008;72(5):847–65. doi: 10.1093/poq/nfn063

[3] Ye C, Fulton J, Tourangeau R. More positive or More Extreme? A Meta-Analysis of Mode Differences in Response Choice. Public Opinion Q. 2011;75(2):349–65. doi: 10.1093/poq/nfr009

[4] Tourangeau R, Yan T. Sensitive questions in surveys. Psychol Bull. 2007;133(5):859–83. doi: 10.1037/0033-2909.133.5.859 17723033

[5] Villar A, Fitzgerald R. Using mixed modes in survey research: Evidence from six experiments in the ESS. In: Breen MJ, editor. Values and Identities in Europe: Evidence from the European Social Survey. 2017. p. 273–310. doi: 10.4324/9781315397146

[6] De Leeuw ED. Data quality in mail, telephone and face to face surveys. ERIC; 1992. Available from: http://eric.ed.gov/?id=ED374136

[7] Vannieuwenhuyze J, Loosveldt G, Molenberghs G. A Method for Evaluating Mode Effects in Mixed-mode Surveys. Public Opin Q. 2010;74(5):1027–45. doi: 10.1093/poq/nfq059

[8] Heerwegh D, Loosveldt G. Face-to-Face versus Web Surveying in a High-Internet-Coverage Population: Differences in Response Quality. Public Opin Q. 2008;72(5):836–46. doi: 10.1093/poq/nfn045

[9] Braekman E, Berete F, Charafeddine R, Demarest S, Drieskens S, Gisle L, et al. Measurement agreement of the self-administered questionnaire of the Belgian Health Interview Survey: Paper-and-pencil versus web-based mode. PLoS One. 2018;13(5):e0197434. doi: 10.1371/journal.pone.0197434 29782504 PMC5962098

[10] Shino E, Martinez MD, Binder M. Determined by Mode? Representation and Measurement Effects in a Dual-Mode Statewide Survey. J Surv Stat Methodol. 2021;10(1):183–202. doi: 10.1093/jssam/smab012

[11] Mavletova A, Couper MP. Sensitive topics in PC web and mobile web surveys: Is there a difference? Surv Res Methods. 2013;7:191–205.

[12] Chang L, Krosnick JA. National Surveys Via Rdd Telephone Interviewing Versus the Internet. Public Opin Q. 2009;73(4):641–78. doi: 10.1093/poq/nfp075

[13] Tourangeau R, Rips LJ, Rasinski K. The Psychology of Survey Response. Cambridge: Cambridge University Press; 2000.

[14] Krosnick JA, Alwin DF. An Evaluation of a Cognitive Theory of Response-Order Effects in Survey Measurement. Public Opin Q. 1987;51(2):201–19. doi: 10.1086/269029

[15] Galesic M, Tourangeau R, Couper MP, Conrad FG. Eye-Tracking Data: New Insights on Response Order Effects and Other Cognitive Shortcuts in Survey Responding. Public Opin Q. 2008;72(5):892–913. doi: 10.1093/poq/nfn059 21253437 PMC3022327

[16] Holbrook AL, Krosnick JA, Moore D, Tourangeau R. Response order effects in dichotomous categorical questions presented orally the impact of question and respondent attributes. Public Opin Q. 2007;71(3):325–48. doi: 10.1093/poq/nfm024

[17] Slomczynski KM, Tomescu-Dubrow I, Jenkins JC, Wolf C. Objectives and Challenges of Survey Data Harmonization. In: Slomczynski KM, Tomescu-Dubrow I, Jenkins JC, Wolf C, editors. Survey Data Harmonization in the Social Sciences. Wiley; 2024. p. 1–20. Available from: https://onlinelibrary.wiley.com/doi/

[18] Fitzgerald R. Responding to the pandemic: A 3 country self-completion push to web experiment. Youtube; 2021. Available from: https://www.youtube.com/watch?v=1zyS3WU3tjE

[19] Hanson T, Fitzgerald R, Comanaru R. Self-Completion Study Based on the 2021 European Social Survey in Great Britain. [data collection]. UK Data Service; 2024. doi: 10.5255/UKDA-SN-9038-1

[20] Hanson T, Aizpurua E, Fitzgerald R, Vukovic M. Surely shorter is better? A questionnaire length experiment in a self-completion survey. In: European Social Survey Conference. 2024. Available from: https://europeansocialsurvey.org/sites/default/files/2024-09/Surely_shorter_isbetter_ESS_Conference.pdf

[21] Kern C, Klausch T, Kreuter F. Tree-based Machine Learning Methods for Survey Research. Surv Res Methods. 2019;13(1):73–93. doi: 10.18148/srm/2019.v13i1.7395 32802211 PMC7425836

[22] Kim H, Markus HR. Deviance or uniqueness, harmony or conformity? A cultural analysis. J Pers Soc Psychol. 1999;77(4):785–800. doi: 10.1037/0022-3514.77.4.785

[23] Johnson TP, Shavitt S, Holbrook AL. Survey Response Styles Across Cultures. In: Matsumoto D, van de Vijver FJR, editors. Cross-Cultural Research Methods in Psychology. Cambridge University Press; 2011.

[24] Ke G, Meng Q, Finley T, Wang T, Chen W, Ma W, et al. LightGBM: A Highly Efficient Gradient Boosting Decision Tree. In: 31st International Conference on Neural Information Processing Systems. 2017. doi: 10.1109/ICCSE.2019.8845529

[25] Twala BETH, Jones MC, Hand DJ. Good methods for coping with missing data in decision trees. Pattern Recognit Lett. 2008;29(7):950–6. doi: 10.1016/j.patrec.2008.01.010

[26] Morren M, Gelissen JPTM, Vermunt JK. Dealing with extreme response style in cross-cultural research: a restricted latent class factor analysis approach. Sociol Methodol. 2011;41:13–47.

[27] Lugtig P. Round 9–10 comparison [PDF]. In: European Social Survey [Internet]. 2024 [cited 12 Jun 2025]. Available from: https://www.europeansocialsurvey.org/sites/default/files/2024-10/round-9-10-comparison-final.pdf

[28] Lugtig P. Comparison of ESS rounds 9 and 10: Mode differences [PDF]. In: European Social Survey [Internet]. 2024 [cited 11 Jun 2025]. Available from: https://www.europeansocialsurvey.org/sites/default/files/2024-10/round-9-10-comparison-final.pdf

[29] Preparing for Round 12 data collection. In: European Social Survey [Internet]. 2025 [cited 10 Jun 2025]. Available from: https://www.europeansocialsurvey.org/news/article/preparing-round-12-data-collection

[30] Stening BW, Everett JE. Response Styles in a Cross-Cultural Managerial Study. J Soc Psychol. 1984;122(2):151–6. doi: 10.1080/00224545.1984.9713475

[31] Chen C, Lee S, Stevenson HW. Response Style and Cross-Cultural Comparisons of Rating Scales Among East Asian and North American Students. Psychol Sci. 1995;6(3):170–5. doi: 10.1111/j.1467-9280.1995.tb00327.x

[32] Marshall R, Lee C. A cross-cultural, between-gender study of extreme response style. Eur Adv Consum Res. 1998;3:90–5.

