# Supplementary material for: Effects of survey administration mode on response profiles are predictable, and robust across countries: Evidence from 29 countries using machine-learning models
Source: PLoS One. 2025 Sep 12;20(9):e0330182. doi: 10.1371/journal.pone.0330182 (PMC12431400; doi:10.1371/journal.pone.0330182)
Supplement: S1 Table — (DOCX) [file pone.0330182.s001.docx]

**Table S1**

*Variables Used in Study.*

|  | Variable name | Label |
| --- | --- | --- |
| 1 | essround | ESS round |
| 2 | cntry | Country |
| 3 | nwspol | News about politics and current affairs, watching, reading or listening, in minutes |
| 4 | netusoft | Internet use, how often |
| 5 | ppltrst | Most people can be trusted or you can't be too careful |
| 6 | pplfair | Most people try to take advantage of you, or try to be fair |
| 7 | pplhlp | Most of the time people helpful or mostly looking out for themselves |
| 8 | polintr | How interested in politics |
| 9 | psppsgva | Political system allows people to have a say in what government does |
| 10 | actrolga | Able to take active role in political group |
| 11 | psppipla | Political system allows people to have influence on politics |
| 12 | cptppola | Confident in own ability to participate in politics |
| 13 | trstp | Trust in country's parliament |
| 14 | trstlgl | Trust in the legal system |
| 15 | trstplc | Trust in the police |
| 16 | trstplt | Trust in politicians |
| 17 | trstprt | Trust in political parties |
| 18 | trstep | Trust in the European Parliament |
| 19 | trstun | Trust in the United Nations |
| 20 | prtdgcl | How close to party |
| 21 | lrscale | Placement on left right scale |
| 22 | stflife | How satisfied with life as a whole |
| 23 | stfeco | How satisfied with present state of economy in country |
| 24 | stfgov | How satisfied with the national government |
| 25 | stfdem | How satisfied with the way democracy works in country |
| 26 | stfedu | State of education in country nowadays |
| 27 | stfhlth | State of health services in country nowadays |
| 28 | gincdif | Government should reduce differences in income levels |
| 29 | freehms | Gays and lesbians free to live life as they wish |
| 30 | hmsfmlsh | Ashamed if close family member gay or lesbian |
| 31 | hmsacld | Gay and lesbian couples right to adopt children |
| 32 | euftf | European Union: European unification go further or gone too far |
| 33 | impcntr | Allow many/few immigrants from poorer countries outside Europe |
| 34 | imbgeco | Immigration bad or good for country's economy |
| 35 | imueclt | Country's cultural life undermined or enriched by immigrants |
| 36 | imwbcnt | Immigrants make country worse or better place to live |
| 37 | happy | How happy are you |
| 38 | sclmeet | How often socially meet with friends, relatives or colleagues |
| 39 | inprdsc | How many people with whom you can discuss intimate and personal matters |
| 40 | sclact | Take part in social activities compared to others of same age |
| 41 | aesfdrk | Feeling of safety of walking alone in local area after dark |
| 42 | health | Subjective general health |
| 43 | atchctr | How emotionally attached to [country] |
| 44 | atcherp | How emotionally attached to Europe |
| 45 | rlgdgr | How religious are you |
| 46 | rlgatnd | How often attend religious services apart from special occasions |
| 47 | pray | How often pray apart from at religious services |
| 48 | hincfel | Feeling about household's income nowadays |
