# Supplementary material for: Effects of survey administration mode on response profiles are predictable, and robust across countries: Evidence from 29 countries using machine-learning models
Source: PLoS One. 2025 Sep 12;20(9):e0330182. doi: 10.1371/journal.pone.0330182 (PMC12431400; doi:10.1371/journal.pone.0330182)
Supplement: S10 Fig — (DOCX) [file pone.0330182.s011.docx]

**Figure S10**

*Feature Importance based on gain, for the classification task of classes SC-10 vs. SC-89, within Sweden.*
